# Complementary Yet Distinct Roles of GLP-1 Receptor Agonists and SGLT2 Inhibitors in Cardiovascular Risk Reduction

**DOI:** 10.3390/biomedicines13112595

**Published:** 2025-10-23

**Authors:** Nóra Homoródi, Éva Varga, Zoltán Szabó, Ferenc Sztanek, Mariann Harangi

**Affiliations:** 1 Department of Cardiology, Faculty of Medicine, University of Debrecen, 4032 Debrecen, Hungary; 2 Department of Internal Medicine and Hematology, Semmelweis University, 1085 Budapest, Hungary; 3 Department of Emergency Medicine, Faculty of Medicine, University of Debrecen, 4032 Debrecen, Hungary; 4 Division of Metabolism, Department of Internal Medicine, Faculty of Medicine, University of Debrecen, 4032 Debrecen, Hungary; 5 Institute of Health Studies, Faculty of Health Sciences, University of Debrecen, 4032 Debrecen, Hungary; 6 ELKH-UD Vascular Pathophysiology Research Group 11003, University of Debrecen, 4032 Debrecen, Hungary

**Keywords:** glucagon-like peptide-1 receptor inhibitors, sodium–glucose transport protein 2 inhibitors, cardiovascular diseases, atherosclerosis, type 2 diabetes mellitus, therapeutic inertia, adverse effects, personalized treatment

## Abstract

Novel antidiabetic drugs introduced in the last decade have not only revolutionized the treatment of type 2 diabetes mellitus but have also changed our cardiovascular risk reduction strategy. Glucagon-like peptide-1 (GLP-1) receptor agonists reduce the risk of atherosclerotic diseases primarily through their complex anti-atherosclerotic effect due to their endothelial function-improving, anti-inflammatory, anti-thrombotic, and plaque-stabilizing effects. Sodium–glucose cotransporter 2 (SGLT2) inhibitors, on the other hand, have a favorable cardiovascular effect, mainly by increasing sodium excretion, reducing plasma volume, enhancing the use of ketone bodies as metabolic substrates in heart and kidney tissues, and reducing oxidative stress and uric acid serum levels. However, when using these two groups of drugs, important questions arise. What criteria should be used to decide on the administration of one or the other class of drugs? Which group of agents can be used more effectively to reduce our patients’ cardiovascular risk? What are the possible adverse effects? What can be gained by combining the two drugs? Our objective was to provide a current literature-based and comparative summary on the mechanisms of action, cardiovascular-risk-reducing efficacy, and safety profiles of these two drug classes, with an emphasis on identifying key factors influencing everyday clinical decision-making.

## 1. Introduction

Cardiovascular diseases (CVDs), including coronary artery disease, myocardial infarction, ischemic stroke, and peripheral arterial disease, have been leading causes of death worldwide for decades [1]. This is primarily due to the high prevalence of risk factors such as obesity, hypertension, hyperlipidemia, smoking, and type 2 diabetes mellitus (T2DM) [2]. The use of medications for cardiovascular prevention has significantly reduced the risk of these conditions. Although agents such as angiotensin-converting enzyme inhibitors, angiotensin receptor blockers, statins, sacubitril, aldosterone antagonists, and antiplatelet drugs have proven to be effective, the so-called cardiovascular residual risk remains substantial even when these drugs are used in combination [3]. Therefore, the discovery of any new class of drugs providing further risk reduction is of utmost importance. Glucagon-like peptide-1 (GLP-1) receptor agonists (GLP-1 RAs) and sodium–glucose cotransporter 2 (SGLT2) inhibitors—originally developed for the treatment of T2DM—have undergone mandatory cardiovascular safety trials during their development. These trials demonstrated significant cardiovascular risk-reducing efficacy for many agents in these two drug classes, in both primary and secondary prevention settings [4,5,6,7,8]. GLP-1 receptor agonists reduce the incidence of events related to atherogenic cardiovascular disease, particularly ischemic stroke, and improve renal outcomes. However, their effect on the incidence and severity of heart failure appears to be limited. In contrast, the most prominent protective effects of SGLT2 inhibitors are a substantial reduction in hospitalization for heart failure and a notable slowing of chronic kidney disease progression [9]. Subsequent large-scale, multicenter randomized trials have confirmed the cardiovascular risk-lowering benefits of these drugs in nondiabetic populations as well, including patients with obesity, heart failure, and chronic kidney disease [10].

Currently, more than 500 million people worldwide are living with T2DM, associated with a significantly increased risk of cardiovascular disease [11]. This patient population might be the one that may derive the greatest benefit from treatment with GLP-1 RAs and SGLT2 inhibitors. Indeed, a previous meta-analysis of eight cardiovascular outcome trials that included data from 60,080 participants (72.4% with established CVD) demonstrated that GLP-1 RA therapy can lower the risk of major cardiovascular events (MACEs) by 14%, cardiovascular death by 13%, non-fatal stroke by 16%, hospitalization for heart failure (HF) by 10%, all-cause mortality by 12%, and macroalbuminuria by 26% among individuals with T2DM [12]. These findings have reshaped the strategy for cardiovascular risk reduction in T2DM, and today, these drugs are incorporated into international clinical guidelines as effective tools for reducing cardiovascular risk [13]. However, the mechanisms of action of the two drug classes differ fundamentally, and there are also significant variations in efficacy between individual agents within each class. Therefore, in everyday clinical practice, it is crucial to be aware of all factors that may influence therapeutic decisions for a given patient.

Based on the available literature, our objective was to provide an overview and compare GLP-1 RAs and SGLT inhibitors regarding mechanisms of action, potential adverse effects, and ability to modify or prevent cardiovascular comorbidities associated with T2DM. Furthermore, we aimed to explore the potential reasons for existing therapeutic inertia and the benefits of their combined use, all from the perspective of the practicing clinician. We aimed to compile a clinically oriented review that supports everyday medical practice by offering practical guidance for healthcare professionals involved in the management of patients with T2DM. Although the beneficial effects of these agents are well known in other comorbidities (e.g., renal disease and metabolic fatty liver), our work primarily focuses on their impact on cardiovascular risk reduction.

## 2. GLP-1 Receptors and GLP-1 Receptor Agonists

### 2.1. The Physiological Role of GLP-1 and Its Receptors in the Functioning of the Cardiovascular System

GLP-1 receptor expression has been demonstrated in a wide range of organs and tissues, with the highest levels found in the beta cells of the pancreatic islets of Langerhans [14] and in specific regions of the central nervous system, including the amygdala, nucleus accumbens, and substantia nigra [15]. In the heart, GLP-1 is expressed in both atrial and ventricular tissues, including the sinoatrial node [14,16], and the presence of its receptors has also been detected on the surface of endothelial cells in blood vessels [17].

GLP-1 was first described in 1987 as an incretin hormone produced by the gastrointestinal tract after food intake that stimulates insulin secretion in a glucose-dependent manner [14,15]. Its beneficial effects on appetite regulation, energy intake, and body weight were identified later, in 1998 [16]. Similar to other incretin hormones, GLP-1 is present in the plasma at very low concentrations during fasting [17]. However, its production significantly increases after food intake, primarily in the L-cells of the small intestine—particularly the lower part of the jejunum—as well as in the colon and rectum. This production is mainly stimulated by carbohydrates and triglycerides absorbed during digestion and their breakdown products and, to a lesser extent, by proteins and amino acids. Among amino acids, glutamine has been shown to be a particularly strong stimulator of GLP-1 secretion [18]. GLP-1 concentrations begin to rise approximately 10–15 min after eating, reaching peak levels between 45 and 90 min post-meal. The stimulation of GLP-1 production triggered by food intake and absorption may also be mediated by glucose-dependent insulinotropic peptide (GIP), produced in the upper gastrointestinal tract, along with other enteroendocrine hormones [19] and neurotransmitters via the intramural plexus [20].

### 2.2. GLP-1 Receptor Agonists’ Mechanism of Action

Under physiological conditions, GLP-1 is rapidly degraded within minutes by dipeptidyl peptidase-4 (DPP4). In contrast, GLP-1 RAs are resistant to DPP4-mediated degradation, resulting in significantly prolonged half-lives compared to native GLP-1. Sustained and meal-independent activation of the GLP-1 receptor enables prolonged glycemic control, appetite regulation, and continuous direct cardiovascular effects. Most GLP-1 RA-based therapies are administered via subcutaneous injection: twice daily (exenatide), once daily (lixisenatide and liraglutide), or once weekly (exenatide once weekly, dulaglutide, albiglutide, and semaglutide) [21]. The first oral formulation is oral semaglutide, which has demonstrated efficacy comparable to that of injectable GLP-1 RAs [22,23]. In addition to pure GLP-1 RAs, the first dual GLP-1/GIP receptor agonist, tirzepatide, has also been introduced. Furthermore, the development of several other dual- and triple-receptor agonist/antagonist therapies is currently underway (Table 1) [24].

**Figure 1:**
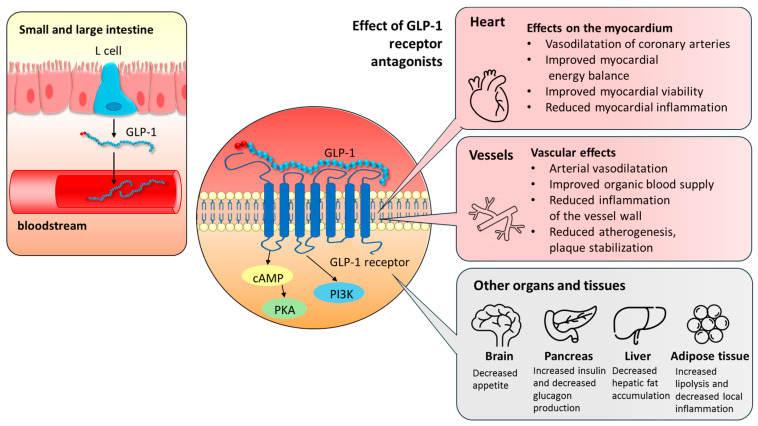
The mechanism of action of GLP-1 receptor agonists with special emphasis on their cardio-vascular effects. GLP-1, secreted by L-cells of the small and large intestine, exerts its effects by entering the circulation and acting on cells that express the GLP-1 receptor, thereby modulating multiple intracellular signaling pathways. The resulting actions are beneficial for myocardial perfusion, energy homeostasis, and viability, as well as for the vascular function of the arterial wall. In addition, GLP-1 has anti-inflammatory and anti-atherosclerotic effects. Its pleiotropic actions on various other organs and tissues indirectly contribute to cardiovascular risk reduction. cAMP: cyclic adenosine monophosphate; GLP-1: glucagon-like peptide-1; PKA: protein kinase A; PI3K: phosphatidylinositol 3-kinase.

Figure 1 summarizes the role of GLP-1 receptors and the mechanism of action of GLP-1 receptor agonists, focusing on their cardiovascular effects.

## 3. The SGLT2 Transporter and Its Inhibitors

### 3.1. The Physiological Role of the SGLT2 Transporter in the Function of the Cardiovascular System

**Figure 2:**
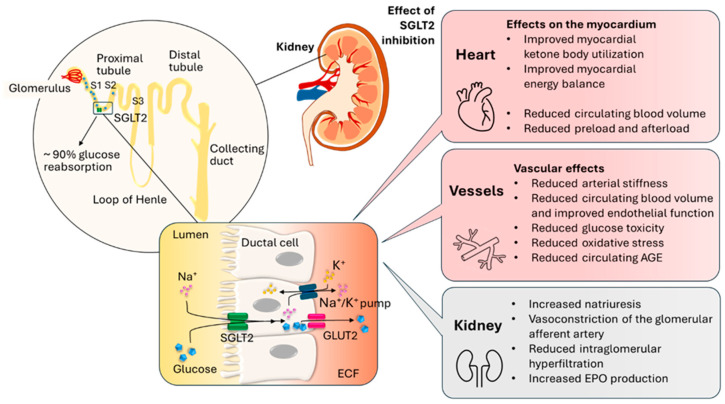
The mechanisms of action of SGLT2 inhibitors with special emphasis on their cardiovascular effects. The SGLT2 transporter protein is responsible for approximately 90% of glucose reabsorption in the proximal tubules of the kidney. Its inhibition leads to increased urinary excretion of glucose and sodium, which results in a reduction in circulating blood volume, as well as decreased preload and afterload. Additionally, myocardial energy balance is improved, along with enhanced utilization of ketone bodies. The beneficial vascular effects are partially attributable to volume reduction, as well as decreased glucotoxicity, oxidative stress, AGEs, and arterial stiffness. Renal effects, including enhanced natriuresis, vasoconstriction of afferent glomerular arterioles, and reduced intraglomerular hyperfiltration, contribute indirectly to cardiovascular benefits. Furthermore, increased EPO production may also play a role in improving cardiovascular function. AGE: advanced glycation end product; GLUT2: glucose transporter 2; ECF: extracellular fluid; EPO: erythropoietin; K^+^: potassium; Na^+^: sodium; S1–S3: segment 1–3; SGLT2: sodium–glucose cotransporter 2.

In healthy individuals, nearly all filtered glucose is reabsorbed, so glucose does not appear in the urine. Glucose reabsorption is partly mediated by glucose transporter proteins (GLUT) and partly through an active transport mechanism involving SGLT proteins, which are also involved in the transport of amino acids, certain ions, and vitamins alongside glucose [25]. SGLTs comprise a large protein family. Among them, the low-capacity isoform SGLT1 is present not only in the small intestine but also in the trachea, prostate, testes, skeletal muscle, heart, and certain regions of the brain (such as the hippocampus), as well as in the kidneys. In contrast, the high-capacity SGLT2 isoform is found almost exclusively in segment 1 of the proximal tubule of the kidneys, where it is responsible for 90% of total glucose reabsorption. It is also in much lower concentrations in the thyroid gland, heart, liver, and brain. Other members of the SGLT family, such as SGLT3, are widely distributed throughout the body and function mainly as glucose sensors, while the roles of other forms (SGLT4, SGLT5, and SGLT6) remain poorly understood in humans [26] (Figure 2).

Approximately 80–90% of filtered glucose is reabsorbed in the S1 segment of the proximal tubule by the SGLT2 protein, while the remaining 10–20% is reabsorbed in the S2 segment via SGLT1. In diabetes, due to increased SGLT2 expression, the amount of reabsorbed glucose significantly rises. However, even with complete SGLT2 inhibition, around 50–60% of filtered glucose is still reabsorbed due to the enhanced activity of SGLT1 proteins [27,28,29]. In both proteins, the energy required for active glucose transport is provided by the Na^+^/K^+^ ATPase pump located in the basolateral membrane of the proximal tubule. Following active transport, glucose exits into the interstitial space and then into the peritubular capillaries through glucose transporter (GLUT) proteins. Specifically, SGLT2 works in conjunction with GLUT2, while SGLT1 functions alongside GLUT1 [26]. Due to this mechanism, glucose reabsorption is coupled with sodium (Na^+^) reabsorption. The increased Na^+^ reabsorption activates the renin–angiotensin system, which leads to vasodilation of the afferent arteriole and vasoconstriction of the efferent arteriole. This raises intraglomerular pressure and the glomerular filtration rate (GFR), contributing to glomerular damage [30,31].

### 3.2. Mechanism of Action of SGLT2 Inhibitors

Currently, there are three main representatives of selective SGLT2 inhibitors: empagliflozin, dapagliflozin, and canagliflozin (Table 1). Their main mechanism of action is to inhibit SGLT2 in the proximal portion of the nephron, with consequent glycosuria and natriuretic effects, attenuation of glomerular hyperfiltration, improvement of blood pressure levels, and weight reduction [32]. Furthermore, these agents have antioxidant, antifibrotic, and anti-inflammatory actions due to the reduction in glycosylation products and the expression of inflammatory molecules [33].

The cardiorenal benefits of SGLT2 transporter inhibitors, introduced as antidiabetic agents, far exceed the clinical gains expected from their antihyperglycemic effects alone (a 0.6–1% reduction in HbA1c levels). Although the full spectrum of the mechanism of action of SGLT2 inhibitors is likely still not fully understood, their introduction has led to the discovery of several previously unknown physiological effects [34].

## 4. Effect of GLP-1 Receptor Agonists on the Cardiovascular System

Within the cardiovascular system, GLP-1 receptors are significantly expressed in endothelial cells throughout the entire arterial system, including the aorta, coronary arteries, and arteries and arterioles supplying various organs [35]. In the heart, GLP-1 receptors are primarily expressed in atrial cardiomyocytes, especially in the cells of the sinoatrial node. The expression of GLP-1 receptors in ventricular cardiomyocytes is controversial; in this region, it is more likely that GLP-1 receptors expressed by endothelial and vascular smooth muscle cells supplying the myocardium and epithelial cells of the endocardium, as well as certain circulating blood cells, play a more significant role [36,37,38,39]. The effect of physiologically produced GLP-1 is transient, as it is broken down within minutes due to the action of the DPP4 enzyme; thus, its impact on the cardiovascular system is also limited. In contrast, GLP-1 RAs have a much longer half-life, which enables sustained 24 h activation of GLP-1 receptors and explains the emergence of pronounced cardioprotective effects. The effects of GLP-1 RAs on the vascular system are complex; in addition to their antihyperglycemic and weight-reducing effects, their anti-inflammatory, antiatherogenic, and antihypertensive properties also likely contribute to their benefits [40].

### 4.1. Anti-Inflammatory and Anti-Atherogenic Effects

**Figure 3:**
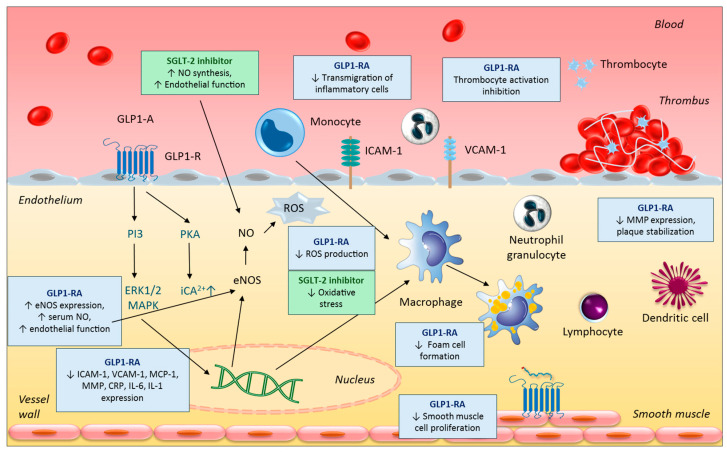
Overview of the key effects of GLP-1 receptor agonists and SGLT2 inhibitors on atherogenesis. Activation of the GLP-1 receptor reduces the expression of multiple inflammatory proteins through the activation of the PI3K and PKA signaling pathways, decreases ROS production, inhibits macrophage foam cell formation, and suppresses the proliferation of vascular smooth muscle cells. Additionally, GLP-1 RA treatment reduces the infiltration of inflammatory cells into the vascular wall, inhibits platelet activation, and exerts plaque-stabilizing effects by suppressing MMP production. SGLT2 inhibition primarily prevents the development and progression of atherosclerosis by improving endothelial function and reducing oxidative stress. CRP: C-reactive protein; eNOS: endothelial nitrogen monoxide synthase; ERK1/2: extracellularly regulated kinase 1/2; GLP1-RA: glucagon-like peptide-1 receptor agonist; iCa^2+^: intracellular calcium; ICAM-1: intercellular adhesion molecule-1; IL-1: interleukine-1; IL-6: interleukine-6; MAPK: mitogen-activated protein kinase; MCP-1: monocyte chemoattractant protein-1; MMP: matrix metalloprotease; NO: nitrogen monoxide; PKA: protein kinase A; PI3 phosphatidylinositol 3; ROS: reactive oxygen species; SGLT-2: sodium–glucose cotransporter 2; VCAM-1: vascular cellular adhesion molecule-1. Upward and downward arrows represent an increase and a decrease in the amount of molecules, respectively.

The anti-inflammatory and anti-atherosclerotic effects of GLP-1 RAs are highly multifaceted [41]. Circulating GLP-1 RAs bind to GLP-1 receptors on the endothelial cell surface, activating adenylate cyclase. This leads to an increase in intracellular cAMP levels, which in turn reduces the expression of monocyte chemoattractant protein-1 (MCP-1), intercellular adhesion molecule-1 (ICAM-1), and vascular cell adhesion molecule-1 (VCAM-1), thereby inhibiting the adhesion and transmigration of myeloid cells into the vascular wall [42]. By reducing the uptake of oxidized LDL, GLP-1 RAs suppress foam cell formation [43]. Additionally, they inhibit vascular smooth muscle cell proliferation and platelet activation, thus reducing intravascular thrombus formation [42]. Through activation of the phosphoinositide 3-kinase (PI3K) signaling pathway, they suppress the expression of various pro-inflammatory cytokines, including interleukin-6 (IL-6), interleukin-1β (IL-1β), C-reactive protein (CRP), and tumor necrosis factor-alpha (TNF-α). Concurrently, by activating endothelial nitric oxide synthase (eNOS), they increase the production of the vasodilator nitric oxide (NO) [44]. A further beneficial effect is the downregulation of matrix metalloproteinase (MMP) expression, which may enhance the stability of existing atherosclerotic plaques [45] (Figure 3).

Among immune system cells, intraepithelial lymphocytes of the gastrointestinal tract express the highest levels of the GLP-1 receptor [46]. Modulation of gastrointestinal immune function is thought to contribute significantly to the favorable alterations in gut microbiome composition observed with GLP-1 RA therapy. However, the impact of these changes on the cardiovascular system is likely indirect [47,48].

### 4.2. Effects on Lipid Parameters

In T2DM, the associated atherogenic dyslipidemia—characterized by elevated triglyceride levels; decreased high-density lipoprotein cholesterol (HDL-C) levels; and an increased proportion of small, dense low-density lipoprotein (LDL) particles—represents a significant risk factor for the development of cardiovascular complications. Therefore, managing these lipid abnormalities is of high importance [49,50]. Reducing total cholesterol and LDL cholesterol (LDL-C) levels is also a key component of cardiovascular risk reduction in T2DM [51]. GLP-1 RAs exert favorable effects on lipid parameters through multiple mechanisms [52]. They have a direct impact on hepatic lipid metabolism by downregulating the expression of several key enzymes involved in lipogenesis, including sterol regulatory element-binding protein-1c (SREBP-1c), fatty acid synthase (FAS), stearoyl-CoA desaturase 1 (SCD1), and acetyl-coenzyme A carboxylase (ACC), thereby reducing de novo hepatic triglyceride synthesis [53]. Moreover, by inhibiting intestinal microsomal triglyceride transfer protein (MTP) and Apolipoprotein B48 (ApoB48) synthesis, GLP-1 RAs attenuate hepatic very low-density lipoprotein (VLDL) secretion [54]. Additionally, liraglutide has been shown to decrease proprotein convertase subtilisin kexin 9 (PCSK9) expression, which contributes to a reduction in plasma cholesterol levels [55]. The enteric nervous system is also likely to be influenced by GLP-1 RA therapy; however, its specific effects on lipid metabolism remain to be elucidated. Activation of the GLP-1 receptor pathway in the central nervous system, via portal afferent vagal fibers, reduces sympathetic nervous system activity, which in turn decreases chylomicron and VLDL secretion, thereby attenuating postprandial hyperlipidemia [56]. According to a recently published meta-analysis including 76 studies and a total of 39,246 patients with T2DM, only PEG-loxenatide was found to significantly increase HDL-C levels, while only semaglutide significantly reduced LDL-C and total cholesterol levels. A significant reduction in triglyceride levels was observed specifically with tirzepatide treatment [57]. Furthermore, a recent one-year follow-up study showed that once-weekly semaglutide administration promotes favorable remodeling of atherogenic lipoprotein subfractions in T2DM [58].

### 4.3. Effects on Body Weight

The beneficial effect of GLP-1 RAs on body weight is well established in patients with T2DM, and this weight reduction also contributes to cardiovascular risk mitigation. The weight-lowering effect is mediated by complex mechanisms [59]. In the central nervous system, GLP-1 RAs suppress appetite, enhance satiety, positively influence food preferences, and increase energy expenditure [60]. Peripherally, they delay gastric emptying [61], improve glucose control by enhancing insulin secretion and reducing glucagon production, and modulate enteric hormonal regulation [62] and gut microbiota composition [63]. Additionally, they reduce adipose tissue inflammation and ectopic fat deposition [64].

According to a meta-analysis that included data from 53 studies, Cagrilintide/semaglutide was identified as having the most potent weight-reducing effect [57]. However, tirzepatide, retatrutide, orforglipron, semaglutide, and liraglutide also induced statistically significant weight loss compared to a placebo [57].

### 4.4. Further Vasculoprotective Effects

Cardiovascular safety trials have reported a moderate increase in heart rate and a variable but clinically significant reduction in blood pressure following GLP-1 RA treatment. In patients with diabetes, this effect is more pronounced, resulting in an additional average reduction of 2–3 mmHg [65].

## 5. Effects of SGLT2 Inhibitors on the Cardiovascular System

The first naturally occurring SGLT inhibitor was phlorizin, which was isolated in 1835 from the bark of the apple tree root by the French chemist C. Petersen. Later, in 1886, the German physician Professor Mering recognized its glucosuric effect [66]. In 1962, Alvarado and Crane demonstrated that the glucosuria was due to phlorizin’s ability to inhibit active glucose transport [67]. Since phlorizin is poorly absorbed from the gastrointestinal tract, intensive research was initiated to develop an orally bioavailable compound. As a result of these efforts, the first SGLT2 inhibitor was synthesized by Japanese scientists in 1996 [66].

The cardiorenal benefits of SGLT2 inhibitors introduced as antidiabetic agents significantly exceed the clinical advantages expected from their antihyperglycemic effects alone, with a 0.6–1% reduction in hemoglobin A1c (HbA1c) levels [68]. Following the initiation of SGLT2 inhibitor therapy, cardiovascular benefits emerge rapidly—within 14 days in patients with HFrEF and within 28 days in those with HFpEF—suggesting the presence of complex mechanisms beyond glycemic control [69]. While the full spectrum of the mechanisms of action of SGLT transporter inhibitors is likely still not completely understood, their introduction has led to the discovery of several previously unrecognized effects.

### 5.1. Body Weight

The urinary excretion of glucose leads to a daily caloric loss of approximately 200–300 kcal, which, based on clinical observations, results in a body weight reduction of 3–5 kg, with considerable inter-individual variation [70]. This initial weight loss is counteracted by the development of compensatory hyperphagia, leading to a plateau in weight reduction after approximately six months. The total weight loss consists of approximately 55–75% body fat (predominantly subcutaneous, with a smaller proportion of visceral fat), 15–35% fluid loss, and around 10% loss of mineral content [71].

### 5.2. Blood Pressure

The natriuretic and glucosuric effects mediated by SGLT2 inhibitors lead to a reduction in preload, as well as pulmonary and systemic congestion [72]. However, the osmotic diuresis induced by SGLT2 inhibitors fundamentally differs from the mechanism of action of conventional diuretics. SGLT2 inhibitors promote the excretion of a larger volume of electrolyte-free water, primarily from the interstitial rather than the intravascular compartment. As a result, congestion is rapidly relieved without significant reductions in systemic blood pressure, arterial filling pressure, or organ perfusion [73]. In addition to reducing preload, SGLT2 inhibitors also decrease afterload, primarily through a 3–5 mmHg reduction in blood pressure and a decrease in arterial stiffness. Notably, the blood pressure and volume reductions associated with SGLT2 inhibitor use are not accompanied by an increase in heart rate, clearly suggesting a suppressive effect on sympathetic nervous system activity. Several in vivo and in vitro studies have demonstrated that the sympatholytic effects of SGLT2 inhibitors are mediated through reduced expression of norepinephrine and tyrosine hydroxylase in the kidneys and heart [74].

### 5.3. Cardiac Metabolism/Metabolic Effects

The administration of SGLT2 inhibitors induces a metabolic state in the body that closely resembles fasting, both at the molecular and cellular levels. These agents are unique among antidiabetic medications in that they enhance both gluconeogenesis and ketogenesis—mechanisms that play a key role in their beneficial effects observed in heart failure and renal insufficiency. A central component of these processes is the activation of silent information regulator 1 (SIRT1), a key regulator of genes involved in energy homeostasis and cellular equilibrium. During fasting, SIRT1 activation promotes ketone body production through increased free fatty acid oxidation and gluconeogenesis [75] (Figure 2).

The downstream effects of SIRT1 activation are mediated via pathways involving fibroblast growth factor 21 (FGF21) and peroxisome proliferator-activated receptor gamma coactivator-1 alpha (PGC-1α). SIRT1, PGC-1α, and FGF21 not only regulate genes and proteins involved in metabolism but also support the function of peroxisomes and mitochondria, thereby contributing to overall energy balance. In addition to these metabolic effects, SGLT2 inhibitors enhance antioxidant enzyme activity, reduce oxidative stress, and attenuate pro-inflammatory processes in both the heart and vasculature. SIRT1 signaling also stimulates autophagy, facilitating the removal of dysfunctional intracellular mitochondria, lysosomes, and other organelles, thereby reducing cellular stress [75]. Furthermore, the reduction in hyperglycemia leads to decreased activation of the renin–angiotensin–aldosterone system (RAAS) and a lower accumulation of advanced glycation end products (AGEs), which collectively reduce myocardial fibrosis, arterial stiffness, and improve diastolic relaxation [76]. Another cardioprotective mechanism of SGLT2 inhibitors involves the direct inhibition of sodium–hydrogen exchanger 1 (NHE1), which results in decreased cytoplasmic Na^+^ and Ca^2+^ levels, while increasing mitochondrial Ca^2+^ concentration—further contributing to improved myocardial function [77].

The effects of SGLT2 inhibitors on lipids may not be clinically significant. Most studies have reported that LDL-C and HDL-C increase and decrease triglyceride levels [78].

## 6. Therapeutic Inertia and the Rationale Behind Concerns Regarding Potential Adverse Effects

Despite clear professional guidelines and the availability of effective pharmacological treatments, glycemic targets are not achieved in nearly half of patients with diabetes [79]. This phenomenon is referred to as therapeutic inertia, a concept not only recognized in diabetes care but also evident in the management of heart failure and hypertension. Clinical inertia was first defined in 2001 as “the failure of healthcare providers to initiate or intensify therapy when indicated.” [80]. In the context of diabetes care, clinical inertia refers to the failure to intensify therapy despite HbA1c levels remaining above target. According to a 2018 review that focused on this issue, on average, more than 1 year (ranging from 0.3 to 7.2 years) elapses before therapeutic intensification occurs in response to suboptimal HbA1c levels [81,82]. Earlier reports indicate that in patients with inadequate glycemic control on metformin monotherapy, no treatment intensification occurred within six months in 38%, 31%, and 28% of cases, despite HbA1c levels exceeding 7.0%, 7.5%, and 8.0%, respectively [83]. Real-world data published in Diabetes Care paint an even more concerning picture. In a cohort of 7438 patients with T2DM, all with HbA1c > 7% and receiving at least two oral antidiabetic agents for over six months, therapy modification was evaluated during a 6-month follow-up period. Among those with HbA1c levels between 7.0 and 7.9%, only 28.4% received treatment intensification. For HbA1c levels between 8.0 and 8.9%, the rate was 46.7%, and even in patients with poorly controlled diabetes (HbA1c > 9%), intensification occurred in just 59.6% of cases [82].

A related but distinct issue is that of reverse therapeutic inertia, in which treatment de-escalation does not occur even when clinically indicated [84]. Until recently, reverse inertia received limited attention, but in 2018, specific recommendations were formulated, stating that physicians should consider step-down therapy in patients with HbA1c levels below 6.5% [85]. Tight glycemic control may be appropriate for younger patients without cardiovascular complications who are receiving modern diabetes management therapies, provided the risk of hypoglycemia is minimal. However, when hypoglycemia-inducing agents are used, achieving an HbA1c level below 6.5% may be hazardous and could also negatively affect patient adherence due to concerns over potential side effects. The possibility of reverse inertia should be considered when a patient with T2DM maintains an HbA1c level below 6.5% for more than 12 months and presents with an elevated risk of hypoglycemia—such as in individuals over 75 years of age, those with impaired renal function, or those with dementia or other cognitive impairments—while being treated with insulin or sulfonylureas [86].

The healthcare system—specifically, the national health insurance provider—defines the conditions for reimbursed prescriptions in the publicly available summary of product characteristics. This regulatory framework restricts the prescribing freedom of physicians, as the 2023 European Society of Cardiology (ESC) guideline, along with its 2024 corrigendum, clearly recommends the use of SGLT2 inhibitors and GLP-1 RAs in patients with established cardiovascular disease or at high cardiovascular risk, regardless of HbA1c levels (Class I, Level A evidence) [87]. In contrast, the current reimbursement policy in Hungary enables subsidized access to these two drug classes only if HbA1c remains > 7% after at least three months of metformin monotherapy combined with lifestyle modification [88].

From the perspective of the healthcare system, prescribing restrictions also pose a challenge for both general practitioners and cardiologists—except in the case of cardiology- or nephrology-specific indications for SGLT2 inhibitors. However, therapeutic inertia is not a phenomenon unique to Hungary; rather, it reflects a complex interplay of factors influenced by country-specific regulations. A Canadian working group conducted a survey among cardiologists to investigate the underlying causes of therapeutic inertia. Their study aimed to explore prescribing patterns and the reasons behind hesitancy to initiate SGLT2 inhibitors and GLP-1 RAs [89].

The responses could be categorized into three main groups:

1. Fear of Adverse Effects:

The most frequently cited concerns included hypoglycemia (both GLP-RAs and SGLT2 inhibitors), urinary tract infections (SGLT2 inhibitors), diabetic ketoacidosis (both GLP-RAs and SGLT2 inhibitors), and other potential side effects [90]. In addition to these, significant apprehension was expressed regarding limited experience in managing such side effects, time constraints in clinical practice, and the financial burden of these therapies [91]. Finally, reluctance due to concerns about polypharmacy also emerged as a notable factor.

2. Evolving Guidelines:

Another major category of responses identified the frequent changes in clinical guidelines as a key factor contributing to therapeutic delay. These changes make it difficult for clinicians to stay current and retain up-to-date knowledge. The complex mechanisms of action of modern antidiabetic agents, as well as changes in their indications and recommended combination therapies, further contribute to uncertainty among physicians in nondiabetology specialties [92].

3. Prescribing Restrictions:

A third group of responses cited the labeling of these agents strictly as antidiabetic drugs, interdisciplinary barriers, and limited access to specialized care centers as primary contributors to therapeutic inertia [93].

4. Patient-Specific Factors:

It is important to emphasize that patients themselves may also play a role in the persistence of therapeutic inertia. Many patients express concerns about potential side effects, lack awareness of target values and their importance, and often do not understand the long-term therapeutic goals. Moreover, financial constraints frequently contribute to their reluctance to initiate or intensify therapy.

Identifying the underlying causes inherently suggests potential solutions. Addressing these causes in reverse order, one of the most important tasks is patient education. This should include explaining the relationship between diabetes complications and poor metabolic control, emphasizing target values and their significance, and highlighting the roles of diet and physical activity. From the perspective of specialized care, education remains crucial, alongside the elimination of interdisciplinary barriers. Additionally, establishing cardiometabolic centers and utilizing the opportunities offered by cardiac rehabilitation programs are essential. With the support of professional organizations and by referencing international and domestic literature, as well as real-world data, efforts should be intensified to advocate for changes in reimbursement and prescribing frameworks.

**Figure 4:**
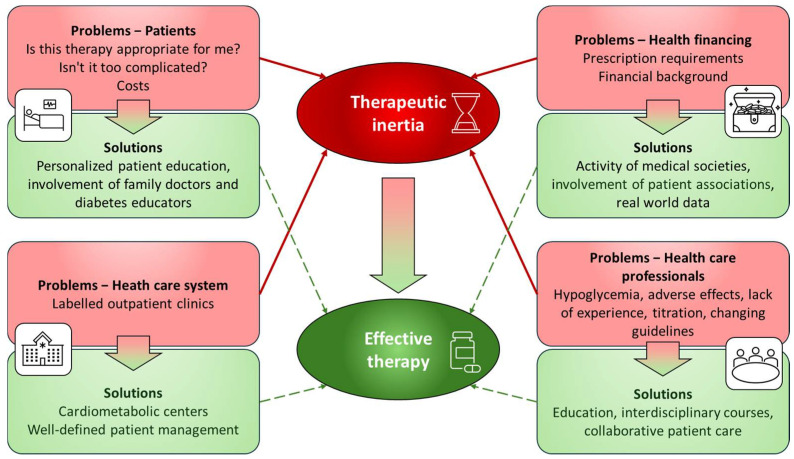
The principal components of therapeutic inertia and potential strategies for its mitigation. Therapeutic inertia is influenced by factors related to the individual patient or healthcare provider, the healthcare system, and reimbursement policies. Accordingly, addressing this issue requires a multifaceted, targeted, and comprehensive approach.

Figure 4 summarizes the principal components of therapeutic inertia and potential mitigation strategies (Figure 4).

### 6.1. Concerns Regarding GLP-1 RA Therapy

A frequently cited issue related to the underutilization of GLP-1 RAs is the need for dose titration, which requires increased attention from both physicians and patients, as well as more frequent clinical visits. In contrast, SGLT2 inhibitors are administered at fixed doses, simplifying their use [94]. Gastrointestinal side effects, including nausea, vomiting, diarrhea, abdominal discomfort, and bloating, are among the most common adverse effects of GLP-1 RAs [95]. These are usually transient and can be successfully managed with gradual dose titration. The association between GLP-1 RA therapy and the risk of acute pancreatitis remains inconclusive. As the condition is rare, it does not pose a significant limitation to the routine clinical use of these agents [95]. Other serious adverse events, including those related to suicidal ideation or behavior, have not been found to occur more frequently during GLP-1 RA therapy [96]. However, it is important to note that the overall benefit–risk ratio of GLP-1 RAs remains highly favorable, particularly given their proven efficacy in reducing cardiovascular events, promoting weight loss, and improving glycemic control [97]. The risk of hypoglycemia is minimal with GLP-1 RA monotherapy or in combination with metformin. However, the risk may increase when these agents are combined with insulin or sulfonylureas, necessitating careful adjustment of concomitant therapeutic doses [98,99]. Reluctance to use injectable therapy could be a barrier, but the availability of oral semaglutide has alleviated this concern for many patients [100]. However, economic considerations and reimbursement restrictions continue to limit access to GLP-1 RAs in some healthcare systems [101,102]. Furthermore, frequently changing reimbursement regulations are challenging for many physicians to navigate, creating potential risks of audits or penalties [103,104]. Nevertheless, practical strategies such as comprehensive patient education, careful titration protocols, and individualized therapy selection may optimize tolerability and adherence, thereby increasing the real-world efficacy of GLP-1 RAs.

### 6.2. Concerns Regarding SGLT2 Inhibitor Therapy

The underutilization of SGLT2 inhibitors is primarily attributed to concerns regarding adverse effects. The most common adverse reactions to SGLT2 inhibitors are genital and urinary tract infections, particularly among women and elderly patients. These infections are usually mild to moderate and can be managed with basic preventive measures, such as good personal hygiene and patient counseling [105,106,107]. With the expansion of the treated patient population, including an increasing number of elderly, multimorbid individuals, reports of euglycemic ketoacidosis have become more frequent. Euglycemic diabetic ketoacidosis, although rare, has been reported, particularly during acute illness, the perioperative period, and prolonged fasting. Appropriate risk-mitigation strategies, such as discontinuing the drug in the event of catabolic states, ensuring adequate hydration, and monitoring patients, can effectively minimize this complication [108]. However, with appropriate caution and adherence to safety guidelines, this complication is generally avoidable [109]. Similar to GLP-1 RAs, SGLT2 inhibitors are associated with a low risk of hypoglycemia, which is clinically relevant only when combined with insulin or insulin secretagogues. In such cases, reducing the dose of the latter agents is recommended [110]. Although the cost of therapy is more favorable compared to GLP-1 RAs, the price of the medication may still limit its initiation in patients with disadvantaged socioeconomic status [102]. Furthermore, prescription regulations also pose practical limitations in routine clinical practice. While cost and the need for a prescription may limit their wider use, the prognostic and preventive benefits of SGLT2 inhibitors clearly outweigh the manageable risks, particularly in patients with concomitant cardiovascular or renal disease.

## 7. Integrated Perspective

Overall, the side effect profiles of GLP-1 RAs and SGLT2 inhibitors are well characterized and predictable. The majority of side effects can be prevented or managed through personalized therapy, dose adjustment, and patient education. When these strategies are employed, the net clinical benefits of these agents, especially when used in combination, significantly outweigh the potential risks, thereby supporting their wider use in contemporary clinical practice.

The key practical considerations for the use of GLP-1 receptor agonists and/or SGLT2 inhibitors are summarized in Table 2.

### 7.1. Criteria for Decision-Making in Clinical Practice

In everyday clinical practice, the decision regarding the optimal therapy for patients with T2DM is influenced by multiple factors, including scientific evidence, clinical guidelines, physician experience, treatment costs, prescribing restrictions, the presence of comorbidities and potential side effects, and the individual preferences of both the physician and the patient [111]. In alignment with professional recommendations, GLP-1 RAs are prioritized in patients with established atherosclerotic cardiovascular disease (such as coronary artery disease, stroke, or peripheral artery disease) and significant excess body weight. In contrast, SGLT2 inhibitors may be preferred in cases of chronic kidney disease or heart failure [13,112]. However, in most patients, these comorbid conditions frequently coexist—even if one predominates clinically—making therapeutic choices more complex. Therefore, when cost and reimbursement considerations allow, the combined use of a GLP-1 RA and an SGLT2 inhibitor should be considered in patients with T2DM. This approach aims to maximize the complementary cardiovascular, renal, and metabolic benefits offered by the two drug classes.

### 7.2. Benefits of Synergy in Metabolic Control and Cardiovascular Prevention

**Figure 5:**
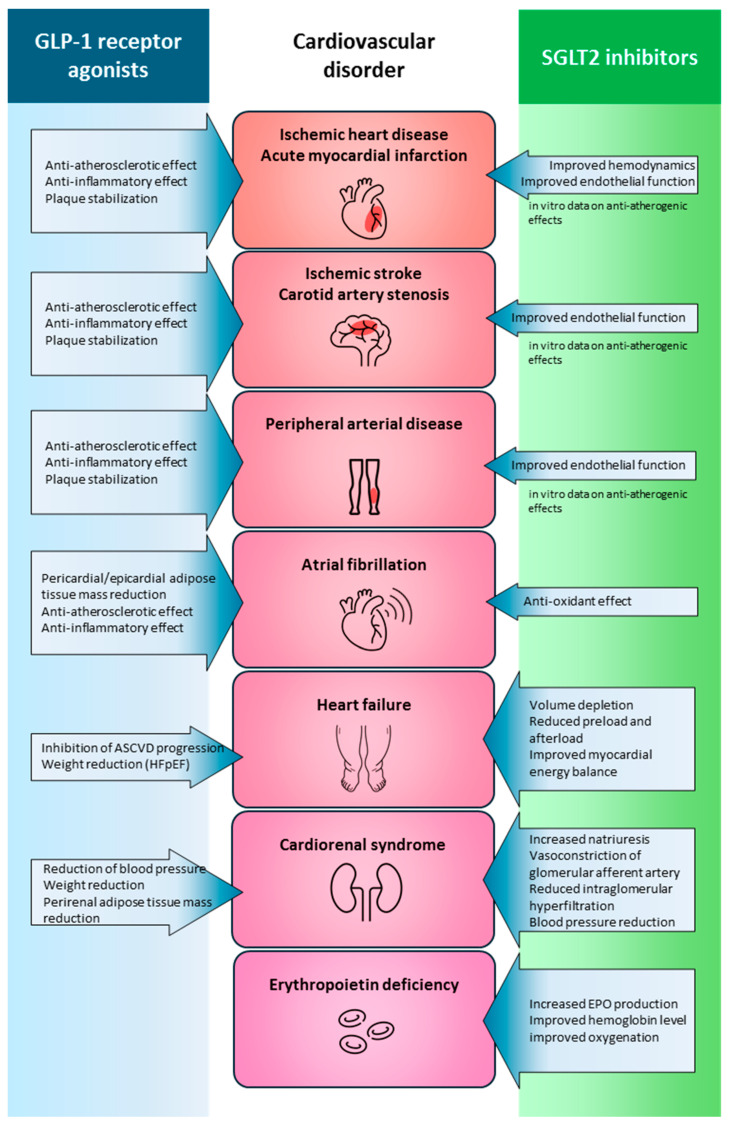
The overview of the beneficial and complementary effects of GLP-1 receptor agonists and SGLT2 inhibitors in cardiovascular diseases. While GLP-1 receptor agonist therapy primarily plays a role in the management of atherosclerotic diseases—including ischemic heart disease, myocardial infarction, ischemic stroke, carotid artery stenosis, and peripheral arterial disease—SGLT2 inhibition is of central importance in the treatment of heart failure and cardiorenal syndrome. Additionally, SGLT2 inhibitors may contribute to cardiovascular risk reduction and the improvement of cardiac output through the stimulation of erythropoietin production. Both drug classes have shown potential benefits in the treatment of atrial fibrillation. The cardiovascular effects of these two therapeutic classes are complementary at multiple target areas. GLP-1: glucagon-like peptide-1; SGLT2: sodium–glucose cotransporter 2.

A comparison of the mechanisms of action of the two drug classes clearly demonstrates that both exert broad and substantial beneficial effects on cardiovascular function [113]. These effects are partly complementary and partly synergistic in nature, providing a strong rationale for their combined use [114] (Figure 5). It has now become evident that the concomitant administration of these two modern therapeutic classes offers significant clinical benefit for patients with T2DM.

To date, three randomized clinical trials have assessed the combination of GLP-1 RA and SGLT2 inhibitor therapy. The DURATION-8 Study was a multicenter, double-blind, randomized controlled trial that aimed to compare the effects of exenatide once weekly plus dapagliflozin, exenatide once weekly alone, or dapagliflozin alone added to metformin monotherapy in patients with T2DM. The study proved that exenatide plus dapagliflozin provided sustained improvements in glycemia, weight, and systolic blood pressure over 52 weeks as well as over 2 years, with no unexpected safety findings [115,116,117].

AWARD-10 was a phase 3b, double-blind, parallel-arm, placebo-controlled, 24-week study on patients with inadequately controlled T2DM taking stable doses of an SGLT2 inhibitor with or without metformin. Based on the results, dulaglutide as an add-on treatment to SGLT2 inhibitors resulted in significant and clinically relevant improvements in glycemic control, with acceptable tolerability that is consistent with the established safety profile of dulaglutide [118].

The SUSTAIN 9 double-blind, parallel-group trial enrolled adults with T2DM and aimed to investigate the efficacy and safety of semaglutide when added to SGLT2 inhibitor therapy. The results showed that adding semaglutide to SGLT2 inhibitor therapy significantly improves glycemic control and reduces body weight in patients with inadequately controlled T2DM and is generally well tolerated [119].

According to the findings of a meta-analysis of eight randomized controlled trials, GLP-1 RA and SGLT2 inhibitor combination therapy showed superior effects in reducing HbA1c, body weight, fasting and postprandial serum glucose levels, systolic blood pressure, body mass index, and LDL-C, without major safety issues, when compared with monotherapy in patients with T2DM [120]. Several further trials and meta-analyses have been published in the last few years with similar conclusions [121,122,123,124,125].

Regarding the reduction in cardiovascular risk, less data is available. However, a meta-analysis of the AMPLITUDE-O and HARMONY Outcomes trials demonstrated that GLP-1 RA therapy reduced the risk of atherosclerotic cardiovascular events and hospitalization due to heart failure, independently of baseline SGLT2 inhibitor use, suggesting an additional risk-reducing effect of the combination therapy [126]. Subgroup analysis from the EXSCEL trial indicated that the addition of exenatide to SGLT2 inhibitor therapy improved cardiovascular outcomes and reduced mortality [127]. Furthermore, a meta-analysis including data from 12 trials showed that the addition of an SGLT2 inhibitor to GLP-1 RA therapy significantly reduced the risk of MACEs [128]. A study conducted in the United Kingdom supports the idea that adding an SGLT2 inhibitor to ongoing GLP-1 RA therapy—or vice versa—significantly reduces the risk of MACEs, as well as severe renal outcomes [129]. Although an increasing use of combination therapy can be observed in patients newly diagnosed with T2DM, it still falls short of the levels recommended by current clinical guidelines [130,131].

According to the available data, combination therapy is safe and well tolerated; the risk of adverse events does not exceed that observed with monotherapy [114].

In patients with T2DM mellitus, both quality of life and long-term prognosis are fundamentally influenced by whether they receive treatment with GLP-1 RAs and/or SGLT2 inhibitors. Despite compelling evidence and clear recommendations in current clinical guidelines, including ADA/EASD Consensus Reports [132] and ESC Guidelines 2023 [87] supporting their use as monotherapy and in combination, these agents remain underutilized in everyday clinical practice. The 2025 ADA/EAS update highlights the significance of the combined use of these two agents in the management of cardio-reno-metabolic (CKM) syndrome, a recommendation further supported by the results of the FLOW trial investigating the renal outcomes of semaglutide treatment [133,134]. In addition, metabolic dysfunction-associated steatohepatitis (MASH) is expected to emerge as a therapeutic target closely linked to cardiorenal–metabolic diseases in the near future [135].

## 8. Conclusions

Based on the above-presented data, the role of GLP-1 RA and SGLT2 inhibitor therapy is well established in reducing cardiovascular risk in patients with type 2 diabetes mellitus. A deeper understanding of the mechanisms of action—particularly their beneficial and complementary effects on the cardiovascular system—as well as insight into potential side effects and strategies to mitigate associated risks, may support a broader adoption of these therapies. Today, these agents are no longer regarded solely as antidiabetic drugs but are increasingly recognized for their anti-atherosclerotic, anti-inflammatory, renoprotective, and cardioprotective properties. This information is of critical importance not only for healthcare professionals but also for policymakers involved in designing reimbursement strategies, as financial accessibility plays a crucial role in widening the implementation of these therapies. Equally essential is the effective communication of their therapeutic benefits to patients and the general public to support informed decision-making and improve adherence. Based on current evidence, the future likely lies in the combined use of GLP-1 RAs and SGLT2 inhibitors. However, further studies are needed to fully explore the potential additional advantages and synergistic effects of combination therapy.

## Figures and Tables

**Table 1:** List of GLP-1 receptor agonists and SGLT2 inhibitors approved by the European Medicines Agency (EMA) and the Food and Drug Administration (FDA).

	EMA-Approved	FDA-Approved
**GLP-1 receptor agonists**	exenatide	exenatide
	lixisenatide	lixisenatide
	semaglutide	semaglutide
	dulaglutide	dulaglutide
	liraglutide	liraglutide
**GLP-1/GIP receptor agonists**	tirzepatide	tirzepatide
**SGLT2 inhibitors**	canagliflozin	canagliflozin
	dapagliflozin	dapagliflozin
	empagliflozin	empagliflozin
		ertugliflozin
		bexagliflozin
**SGLT1/SGLT2 inhibitor**		sotagliflozin

**Table 2:** Quick reference—GLP-1 RAs and SGLT2 inhibitors in the cardiometabolic management of T2DM.

**When to prioritize GLP-1 RAs**
GLP-1 RAs should be considered the preferred option for patients with Established atherosclerotic cardiovascular disease (e.g., coronary artery disease, stroke, peripheral arterial disease). A particular need for stroke prevention. Significant overweight or obesity, given their robust weight-reducing effect. A therapeutic goal of improving lipid profile and exerting anti-atherosclerotic effects.
**When to prioritize SGLT2 inhibitors**
SGLT2 inhibitors are recommended as the first choice in patients with Heart failure (both HFrEF and HFpEF). Chronic kidney disease, with or without albuminuria. The need for early prevention of cardiac decompensation, as benefits emerge within weeks of initiation. A therapeutic goal of volume reduction and blood pressure lowering.
**When to consider combination therapy (GLP-1 RAs + SGLT2 inhibitors)**
The concomitant use of GLP-1 RAs and SGLT2 inhibitors should be considered in Patients with multiple cardiometabolic comorbidities (e.g., T2DM with coexisting CVD and/or CKD). Situations where maximal cardiovascular and renal protection is desired. Patients who fail to achieve glycemic or weight targets with monotherapy. Cases where cost and reimbursement policies do not represent limiting factors.
**General considerations**
Both classes carry a minimal intrinsic risk of hypoglycemia, although the risk increases when combined with insulin or sulfonylureas. GLP-1 RAs: Main adverse events are gastrointestinal; most agents require subcutaneous administration, except for oral semaglutide. SGLT2 inhibitor: Most frequent adverse events are genital infections; rare cases of euglycemic diabetic ketoacidosis may occur under catabolic stress. Both classes have demonstrated reductions in mortality and major cardiovascular events, making them cornerstones of modern cardiometabolic therapy.

## Data Availability

No new data were created or analyzed in this study.

## Abbreviations

The following abbreviations are used in this manuscript:

ACC	Acetyl-coenzyme A carboxylase
AGEs	Advanced glycation end products
ApoB48	Apolipoprotein B48
CKM	Cardio-reno-metabolic
CRP	C-reactive protein
CVDs	Cardiovascular diseases
DPP4	Dipeptidyl peptidase-4
eNOS	Endothelial nitric oxide synthase
ESC	European Society of Cardiology
FAS	Fatty acid synthase
FGF21	Fibroblast growth factor 21
GFR	Glomerular filtration rate
GIP	Glucose-dependent insulinotropic peptide
GLP-1 RA	Glucagon-like peptide-1 receptor agonists
GLP-1	Glucagon-like peptide-1
GLUT	Glucose transporter proteins
HbA1c	Hemoglobin A1c
HDL-C	High-density lipoprotein cholesterol
HF	Heart failure
HFpEF	Heart failure with preserved ejection fraction
HFrEF	Heart failure with reduced ejection fraction
ICAM-1	Intercellular adhesion molecule-1
IL-1β	Interleukin-1β
IL-6	Interleukin-6
LDL	Low-density lipoprotein
LDL-C	LDL cholesterol
MACE	Major cardiovascular event
MASH	Metabolic dysfunction-associated steatohepatitis
MCP-1	Monocyte chemoattractant protein-1
MMP	Matrix metalloproteinase
MTP	Microsomal triglyceride transfer protein
Na^+^	Sodium
NHE1	Sodium–hydrogen exchanger 1
NO	Nitric oxide
PCSK9	Proprotein convertase subtilisin kexin 9
PGC-1α	Peroxisome proliferator-activated receptor gamma coactivator-1 alpha
PI3K	Phosphoinositide 3-kinase
RAAS	Renin–angiotensin–aldosterone system
SCD1	Stearoyl-CoA desaturase 1
SGLT2	Sodium–glucose cotransporter 2
SIRT1	Silent information regulator 1
SREBP-1c	Sterol regulatory element-binding protein-1c
T2DM	Type 2 diabetes mellitus
TNF-α	Tumor necrosis factor-alpha
VCAM-1	Vascular cell adhesion molecule-1
VLDL	Very low-density lipoprotein
